# A statistical network pre-processing method to improve relevance and significance of gene lists in microarray gene expression studies

**DOI:** 10.1186/s12859-022-04936-z

**Published:** 2022-09-27

**Authors:** Giuseppe Agapito, Marianna Milano, Mario Cannataro

**Affiliations:** 1 Department of Law, Economics and Sociology Sciences, University Magna Græcia, 88100 Catanzaro, Italy; 2 Data Analytics Research Center, University Magna Græcia, 88100 Catanzaro, Italy; 3 Department of Medical and Surgical Sciences, University Magna Græcia, 88100 Catanzaro, Italy

**Keywords:** Biological pathways, Differential expressed genes, Pathway enrichment analysis, Statistical analysis, Data mining network, Network analysis, SNPs

## Abstract

**Background:**

Microarrays can perform large scale studies of differential expressed gene (DEGs) and even single nucleotide polymorphisms (SNPs), thereby screening thousands of genes for single experiment simultaneously. However, DEGs and SNPs are still just as enigmatic as the first sequence of the genome. Because they are independent from the affected biological context. Pathway enrichment analysis (PEA) can overcome this obstacle by linking both DEGs and SNPs to the affected biological pathways and consequently to the underlying biological functions and processes.

**Results:**

To improve the enrichment analysis results, we present a new statistical network pre-processing method by mapping DEGs and SNPs on a biological network that can improve the relevance and significance of the DEGs or SNPs of interest to incorporate pathway topology information into the PEA. The proposed methodology improves the statistical significance of the PEA analysis in terms of computed *p* value for each enriched pathways and limit the number of enriched pathways. This helps reduce the number of relevant biological pathways with respect to a non-specific list of genes.

**Conclusion:**

The proposed method provides two-fold enhancements. Network analysis reveals fewer DEGs, by selecting only relevant DEGs and the detected DEGs improve the enriched pathways’ statistical significance, rather than simply using a general list of genes.

**Supplementary Information:**

The online version contains supplementary material available at 10.1186/s12859-022-04936-z.

## Introduction

The advent of microarrays [1] allowed for efficient investigation of genetic matter, making it possible to improve both real-time polymerase chain reaction (RT-PCR) [2] and the Sanger methods [3], allowing large scale studies of differential expressed genes (DEGs) and even single nucleotide polymorphisms (SNPs) [4].

In this manner, microarrays allow for screening of thousands of genes for a single experiment. Sanger-method, RT-PCR and microarrays rely on the extension of small segments of DNA through the *polymerase biological process*. All the cited methods will extend the genetic sequence of interest by adding on the complementary nucleotide from the template DNA strand. These methods allow a relative and accurate quantification of DNA and mRNA molecules with a sufficiently high reproducibility and low variability, and they are all well suited to study gene expression.

However, after the initial fervor, it became apparent that even the lists of DEGs or SNPs were mainly as enigmatic as the first nucleotide sequence of the genome. The main reason being that these lists of DEGs and SNPs are independent from the affected biological context. To overcome this limitation, several statistical software tools [5–9] have been developed to help researchers analyze this enormous amount of microarray data to elucidate more valuable and suitable outcome for clinical activities. In addition, several data mining software tools [10–12] are available that allow computation of multiple associations among SNPs. The produced results, from both categories, provide lists of DEGs or SNPs that are unlikely to be directly used in clinical activities, because results are still disconnected from the affected biological processes.

Pathway enrichment analysis (PEA) can facilitate the interpretation of such a list of DEGs or SNPs, linking both to the affected biological pathways and consequently to the underlying biological functions and processes. Although, PEA can help figure out the affected biological pathways starting from the DEGs or SNPs of interest, poor quality and relevance of the employed input can produce pathways that are not directly related to the condition under investigation. This is due to the fact that, a poor quality list of DEGs or SNPs can enrich a general pathway such as *disease,* rather than a more specific one like *cellular responses to external stimuli*, a well-known pathway involved in the progression of colorectal cancer, for example. These biases prevent researchers from figuring out the proper affected biological pathways and the related functional interactions.

To improve the enrichment analysis results, it is necessary to determine the relevant DEGs that can both improve the *p* value (i.e. relevance) of the enriched pathways and reduce the number of enriched pathways, consequently improving their relevance with respect to the condition under investigation.

For these reasons, we developed a new DEG preprocessing method based on statistical and networks analysis. The proposed method identifies, from the whole DEGs list of interest, the most relevant genes with which to perform PEA. In short, the proposed method follows these steps: *(i)* DEG filtering relies on the *Kruskal–Wallis test* [13] to select only DEGs with similar behaviours from the provided input list, splitting DEGs in up- and down-regulated gene groups. In addition, *Kruskal–Wallis test* returns results in the form of matrices. The provided matrices contain the *p* values for each group, that will be used to build gene interaction networks. In this model, the computed *Kruskal–Wallis* *p* values are considered as a similarity measure among gene pairs [14, 15]. *(ii)* Next, the computed similarity matrices are converted into networks from which the essential DEGs are extracted. *(iii)* Finally, both essential DEGs groups are mapped separately on the human protein-protein interaction (PPI) network obtained from the Integrated Interactions Database (*IID*) database [16] to discover additional relevant genes to perform PEA analysis.

The rest of the paper is organized as follows. Section 2 describes the provenance of the downloaded gene expression data sets, the methods employed to obtain the list of DEGs, and the threshold used to select DEGs. Section 2.6 highlights and details the major phases of the DEG preprocessing methodology. Section 3 describes and discusses the preliminary results as a validation of our approach, highlighting the principal benefits. Section 4 validates the enrichment results by manually exploring the literature and finally, Sect. 5 concludes the paper.

## Methods

### Data set

Microarray assays are extensively used in many omics data analyses for several reasons. First microarrays analyse are cheaper than Next-Generation Sequencing (NGS), RNAseq. Second, extensive microarray studies are available in the literature and cover a variety of different phenotypes. Microarray data are curated, providing well-documented criteria, making it easy to verify the accuracy and reproducibility of the research. In addition, microarray data sets can be used as benchmarks to validate data analysis workflows. Hence, we have chosen to use GEO microarray data sets to perform the preliminary tests of our methodology.

We downloaded from the Gene Expression Omnibus (GEO) database [17] the following data sets:

- **GSE1297** [18] provides microarray correlation analysis of hippocampal gene expression deemed to be responsible for incipient Alzheimer’s disease (AD). The data set contains data from approximately 31 subjects: 9 controls, and 22 cases affected by AD. Expression profiles were collected using Affymetrix Human Genome U133A Array. For further details see https://www.ncbi.nlm.nih.gov/geo/query/acc.cgi?acc=GSE1297.
- **GSE5281** [19–22] contains gene expression profiling data collected from brain samples. The Affymetrix Human Genome U133 Plus 2.0 Array was used to yield the expression profiles. The data set is comprised of data from about 161 subjects: 100 Alzheimer subjects, and 61 controls. Both samples groups are related to six brain regions that are histopathologically or metabolically relevant to AD and aging. For further details see https://www.ncbi.nlm.nih.gov/geo/query/acc.cgi?acc=GSE5281.
- **GSE16759** [23] contains a combination of profiled messenger RNA (*mRNA*) and *microRNA* (*miRNA*) expressions to define the role of miRNAs in AD. Expression profiles were obtained using Affymetrix Human Genome U133 Plus Array and the USC/XJZ Human 0.9 K miRNA-940-v1.0. The overall design of the *GSE16759* data set is parietal lobe tissue from 4 Alzheimer’s subjects and 4 age-matched controls. For further details see https://www.ncbi.nlm.nih.gov/geo/query/acc.cgi?acc=GSE16759.
- **GSE9476** [24] describes the use of microarrays to identify previously unrecognized expression changes that occur only in acute myeloid leukemia (AML) blasts. Expression profiles were obtained using Affymetrix Human Genome U133A Array. The overall design includes gene expression profiles between normal hematopoietic cells from 38 healthy controls, and leukemic blasts from 26 AML patients. Eighteen normal hematopoietic samples included CD34+ selected cells, 10 unselected bone marrows cells, and 10 unselected peripheral blood cells. For further details see https://www.ncbi.nlm.nih.gov/geo/query/acc.cgi?acc=GSE9476.
- **GSE14924** [25] attempts to prove that *T* cells from patients with chronic lymphocytic leukemia (CLL) show differentially regulated genes compared with healthy *T* cells. Expression profiles were obtained using Affymetrix Human Genome U133 Plus 2.0 Array. The overall design includes gene expression profiles of four groups of samples: 10 AML CD4, 10 AML CD8, 10 Healthy CD4, and 11 Healthy CD8. AML samples were chosen to represent the range of prognostic groups and patient outcomes. For further details see https://www.ncbi.nlm.nih.gov/geo/query/acc.cgi?acc=GSE14924.
- **GSE24739** [26, 27] encompasses gene expressions of normal and chronic myelogenous leukemia. The differentially expressed genes were grouped according to their reported functions, and correlations were sought with biological differences previously observed between the same groups. Expression profiles were obtained using Affymetrix Human Genome U133 Plus 2.0 Array. The overall design includes gene expression profiles of 8 AML samples and 4 normal samples. For further details see https://www.ncbi.nlm.nih.gov/geo/query/acc.cgi?acc=GSE24739.

The main features of the six downloaded data sets are listed in Table 1.

**Table 1:** A summarization of the main features of the downloaded data sets

	Disease	Cases	Controls
GSE1297	Alzheimer’s disease	22	9
GSE5281	Alzheimer’s disease	100	61
GSE16759	Alzheimer’s disease	4	4
GSE9476	Acute myeloid leukemia	26	38
GSE14924	Acute myeloid leukemia	20	21
GSE24739	Acute myeloid leukemia	8	4

**Fig. 1:**
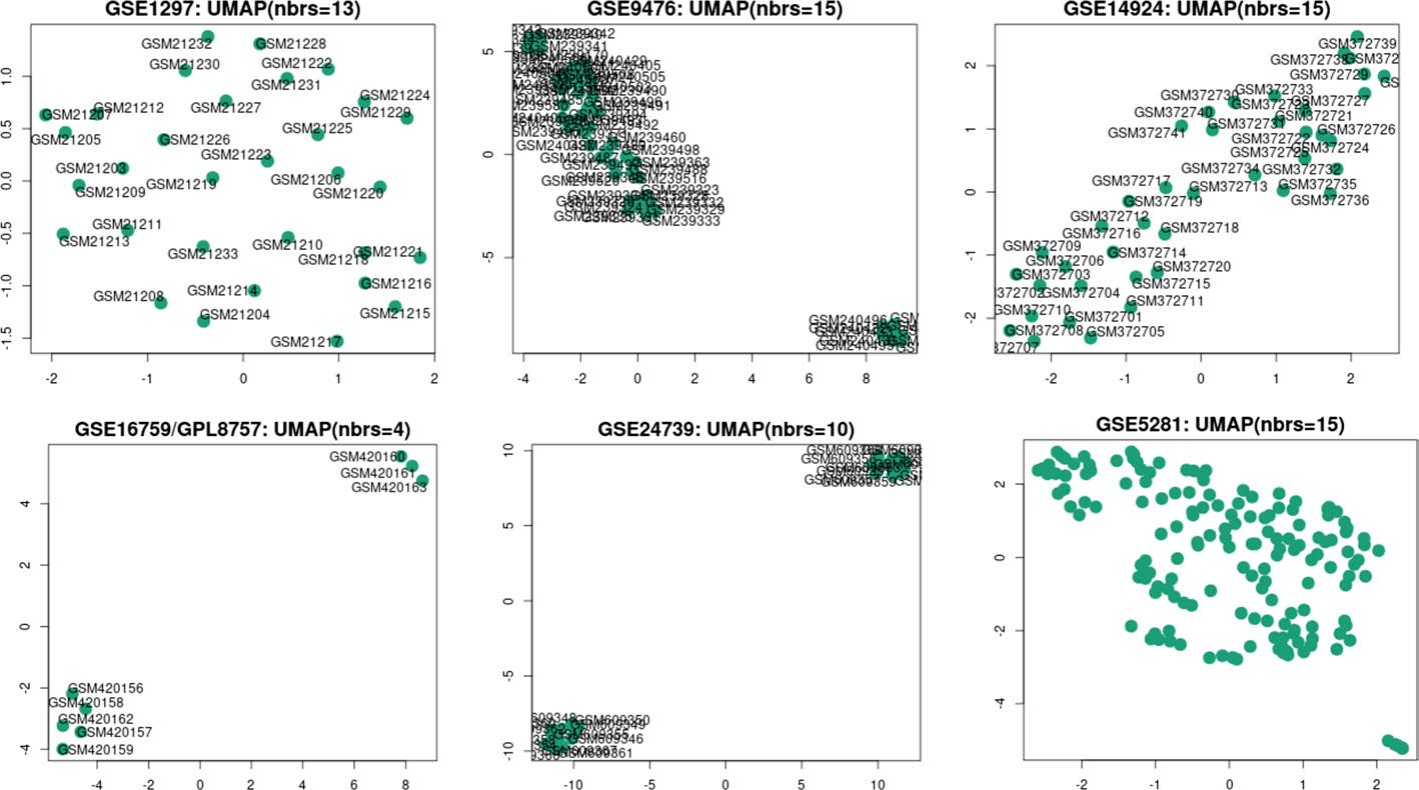
The UMAP of the six downloaded data sets

**Fig. 2:**
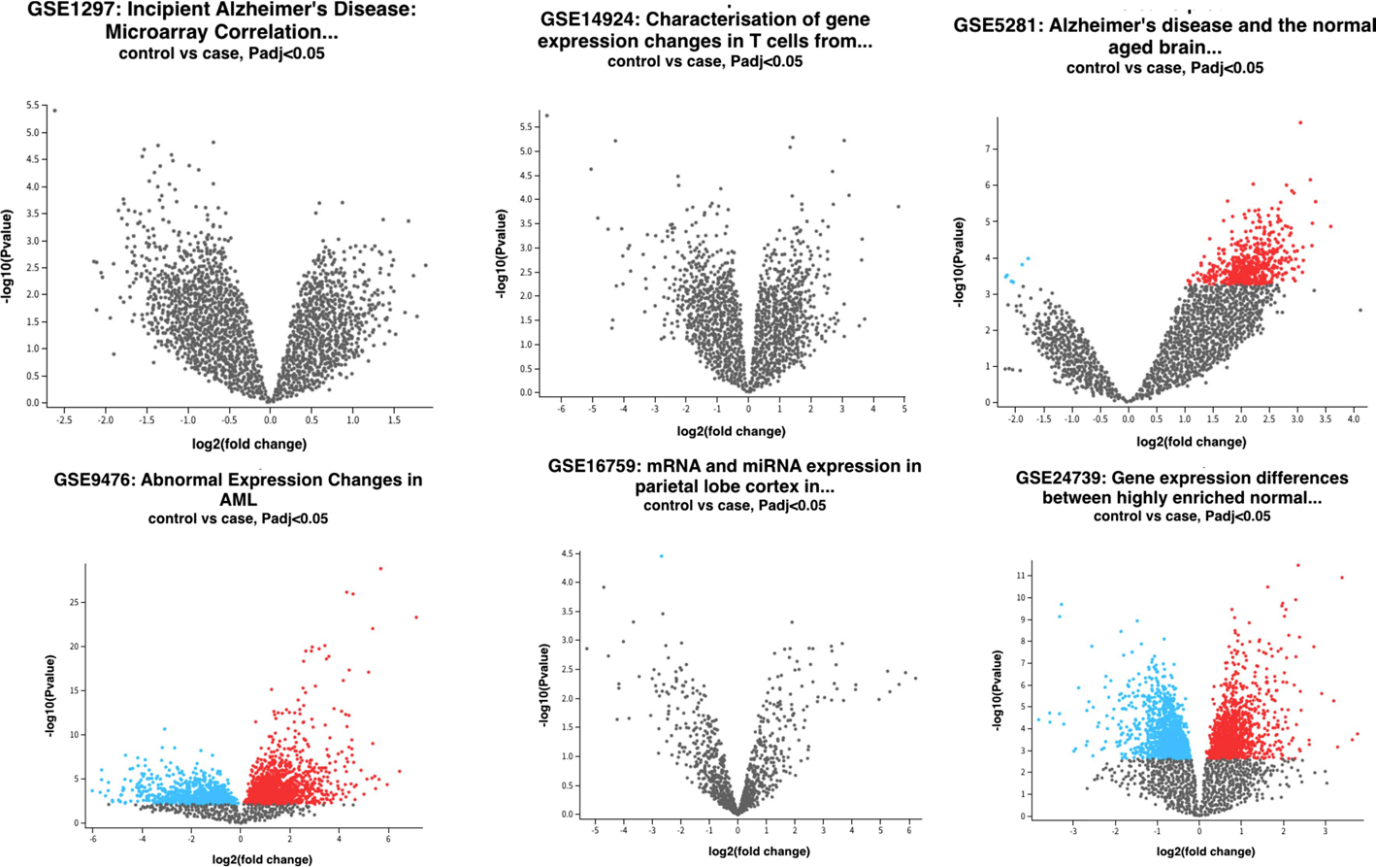
The Volcano Plot of the six downloaded data sets

Figures 1 and 2 show the Uniform Manifold Approximation and Projection (UMAP) and Volcano plot related to the downloaded data sets.

### Detection of DEGs with GEO2R

To identify the differential expressed genes between cases and controls, we used *GEO2R* (https://www.ncbi.nlm.nih.gov/geo/geo2r/). *GEO2R* is an interactive online analysis tool used to detect DEGs enclosed in expression profile array data sets. *GEO2R* allows classification of subjects in several groups, using the *define groups* command. The panel *options* available in *GEO2R*, allow straightforward analysis customization. The option panel enables users to select the statistical corrector, the data normalization method, and the cut-off value to filter out the genes not holding the defined cut-off. In addition, *GEO2R* exploits the limma package to perform inter- and intra-sample normalization. To perform DEG analysis, we selected the false discovery rate (FDR) *p* value adjustment for multiple testing, and the *log data transformation* method to normalize the results. Finally, we selected and downloaded the following results: adjusted *p* value, *p* value, *logFC*, gene symbol, and title.

### Selection of DEGs

A cohort of DEGs was obtained by filtering out all the genes that do not meet the adjusted threshold criteria (*p* value $\le 0.005$ and $|(logFC)| \ge 1.5)$. The fold change logarithm (*logFC*) is a metric to assess the change in the ratio between the expression levels of two genes. Hence, the genes meeting both criteria were designated as DEGs. DEGs associated to negative *logFC* values are classified as down-regulated DEGs, otherwise they were classified as up-regulated DEGs. Next, the DEGs are investigate using the *Kruskal–Wallis* test [13], to differentiate genes with similar behaviour. The Kruskal–Wallis test evaluates the similarity between pairs of genes, assessing if two genes are correlated [14, 15]. In general, Kruskal–Wallis test is applied to test the null hypothesis which states that *k* number of samples have been starved from the same population or an identical population with the same or identical median. In this manner, accepting the null hypothesis, e.g., a *p* value greater than 0.005, allow coupling of genes with the same median among them enabling identification of genes with the same statistical behaviours.

### Pathway enrichment analysis

To identify the connections among DEGs with the affected biological functions, we can use PEA, making it possible to take advantage of the pathway database’s information to discover connections with biological mechanisms. This approach helps researchers interpret gene lists, or other biological entity lists of interest, disconnected from the biological context, facilitating and validating their findings [28, 29]. To perform PEA, we used the BioPAX-Parser (BiP) software tool [30], an automatic and graphics-based tool to achieve PEA by using pathways data encoded in BioPAX format. BioPAX-Parser is fully developed using Java 8, and helps perform PEA by merely loading a list of proteins/genes of interest. Enrichment in BiP implements the Hypergeometric test, False Discovery Rate (FDR), and Bonferroni multiple-test statistical correctors.

### Pathway data

Pathway data were collected from the *Reactome* database [31] (version 79) with BiP. Reactome is an open source, open access, manually curated, and peer-reviewed database of human pathways, biological processes and biochemical reactions. Reactome is the result of the joint efforts of several international research institutes. In the current version, Reactome contains the whole known pathways coming from 22 different organisms including the *Homo sapiens*. Reactome includes over 2, 000 pathways and about 10, 000 annotated proteins for the *Homo sapiens*. Reactome allows to browse pathways through the graphical web interface, as well as download the data in different formats comprising Systems biology markup language (SBML) Level 2, BioPAX Level 2 and Level 3 and other graphical formats for local analysis.

### The DEGs preprocessing method

The proposed statistical network pre-processing methodology automatically determines significant *DEGs* to use in PEA analysis in order to obtain more relevant biological pathways with respect to the condition under investigation. The proposed method consists of the following steps:

1. *Similarity matrix computation*. *Similarity matrices* As a preliminary step, the input DEG list is filtered by using the criteria introduced in Sect. 2.3. The remaining, DEGs are automatically grouped into up- and down- regulated genes, to yield the related up- and down- regulated similarity matrices’ *UpSM* and *DownSM*. The Kruskal–Wallis test [13] is used to compute both the *UpSM* and *DownSM* matrices by using the grouped DEGs. Kruskal–Wallis test is a non-parametric version of a parametric one-way ANOVA with the data substituted by their scores [32]. It works on two or more independent populations whose dimensions, e.g., the number of elements in each population, can be different. Equation 1 shows the formal definition of the Kruskal–Wallis test.
  1 $$\begin{aligned} K = (N-1)\frac{\sum _{i=1}^g n_i(\bar{s}_{i\cdot } - \bar{s})^2}{\sum _{i=1}^g\sum _{j=1}^{n_i}(s_{ij} - \bar{s})^2},\quad \bar{s}_{i\cdot } = \frac{\sum _{j=1}^{n_i}{s_{ij}}}{n_i};\quad \bar{s} =\frac{1}{2} (N+1) \end{aligned}$$
  In Eq. 1, *N* is the total number of elements, *g* is the groups number, $n_i$ is the number of elements in group *i*, $s_{ij}$ is the observation of element *j* from group *i*, $\bar{s}_{i\cdot }$ is the average similarity of all elements in group *i*, and $\bar{s}$ is the average of all the $s_{ij}$ similarities. A generic *SM*’s cell (*i*, *j*) contains the value of the similarity obtained comparing two genes by means of the Kruskal–Wallis test. The Kruskal–Wallis test assesses if two genes are correlated. In this manner, the Kruskal–Wallis test compares the genes with the aim to elucidate statistically similar behaviours among them. Other models [33, 34] used the Wilcoxon test [35, 36] to compute the *SM*. To compute the *SM*, the Wilcoxon test requires that the number of elements in each populations, e.g., the assessed expression levels *n* for each gene is $n \ge 20$. Conversely, the Kruskal–Wallis test works on 2 or more independent populations which may have different number of elements. A lower score (i.e, *p* value) implies that two genes are different according to the *logFC*. Otherwise, a higher score implies that genes show a similarity. The threshold was set to 0.005. Hence, the *SM*’s will contain only *p* values $\ge 0.005$, 0 otherwise.
2. *Converting similarity matrix to network* The *UpSM* and *DownSM* matrices are converted to networks $N_{up}$ and $N_{down}$, where nodes are the genes and the edges connect them when the similarity value among two genes in the (*i*-*th*, *j*-*th*) cell exceeds the similarity threshold (e.g., *p* values $\ge 0.005$). The *Closeness Centrality (CC)* measure determines from the $N_{up}$ and $N_{down}$ networks the genes to include in the respective *Essential Gene sets* $EG_{up}$ and $EG_{down}$. The CC is tightly related to the notion of distance between nodes and indicates how close a node is to all other nodes in the network. It is calculated as the average of the shortest path length from a node to every other node in the network. Only the nodes with CC values less than or equal to the computed average CC (e.g., $CC(n_i)\le CC_{avg}(N)$) were included in the respective $EG_{up}$ or $EG_{down}$ gene sets.
3. *Improving genes relevance* The essential genes in both *EG* sets identified in the previous step are mapped onto the protein-protein interaction (PPI) network obtained from the *IID* [16] database. All DEGs that do not exist in the *iidNetwork* i.e., $N_{iid}$ are filtered out. For each mapped gene from the respective gene sets, $EG_{up}$ and $EG_{down}$, we computed from the $N_{iid}$, the neighborhood with a radius equal to 1, yielding respectively the *up-regulated gene community* $GC_{up}$, and the *down-regulated gene community* $GC_{down}$. In this way, it is possible to identify similar genes, and similar genes tend to interact among them to complete biological tasks. Finally, from both neighborhoods, all the nodes with a *Bottleneck* value greater than the *average Bottleneck* value, were selected to compute PEA.

**Fig. 3:**
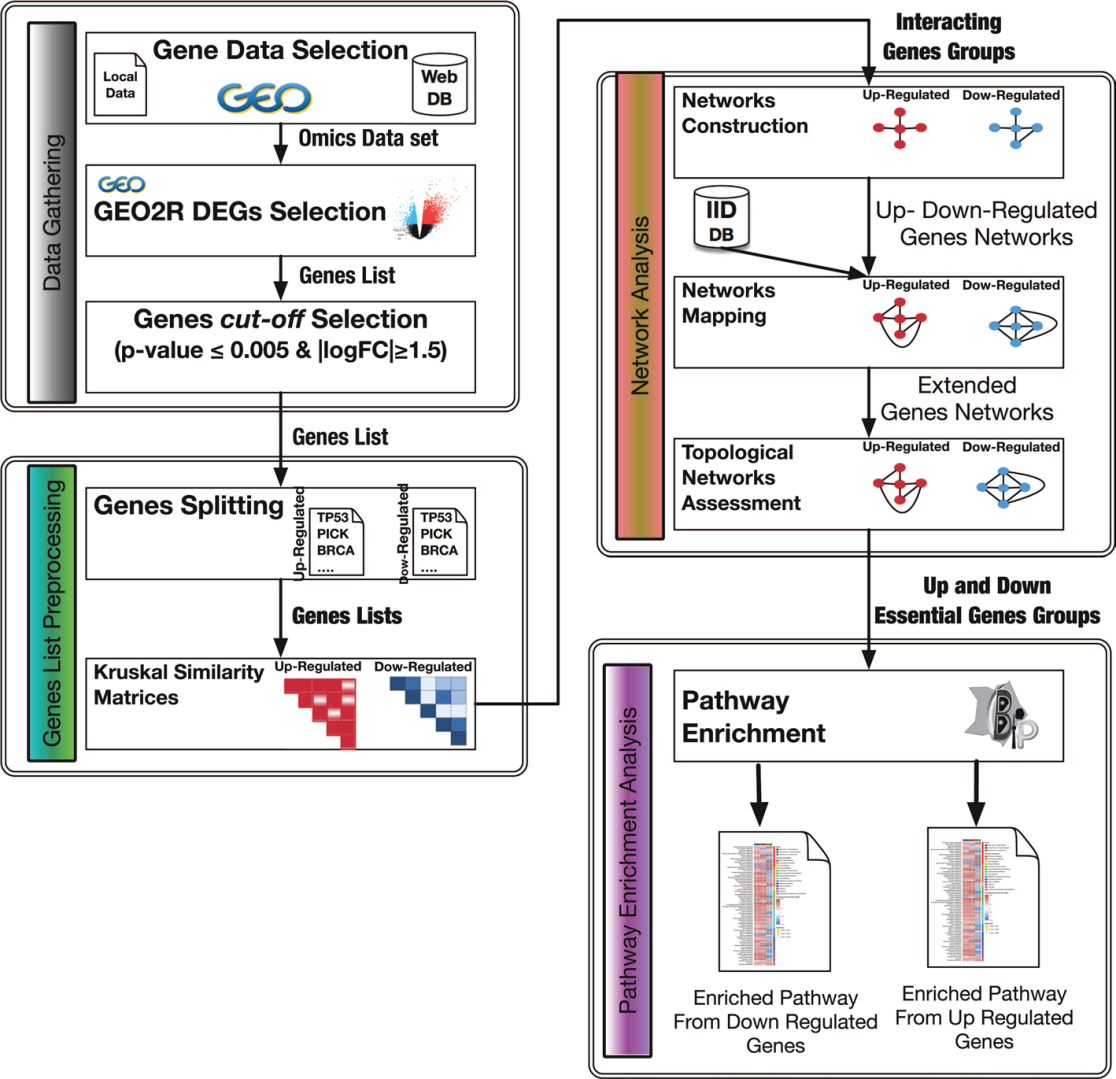
The main steps of the proposed method

Figure 3 shows the main steps of the proposed method.

## Results

The six data sets obtained from GEO and analyzed through the GEO2R framework were used as benchmark data. Table 2 contains the information the preprocessing of the original data sets of Table 1 referring to acute myeloid leukemia and Alzheimer’s disease Analysis of all data sets began by filtering out genes using the threshold values defined Sect. 2.3. Next, the genes holding the threshold criteria were split into up and down-regulated gene sets, $EG_{up}$ and $EG_{down}$. It is worth noting that up- and down-regulated gene sets do not overlap, e.g., $\{EG_{up} \cap EG_{down} = \emptyset \}$. Third, genes in both gene sets were analyzed using the Kruskal–Wallis test to identify genes with the same behavior to compute similarity matrices, *UpSM* and *DownSM*. The *UpSM* and *DownSM* matrices were converted into networks $N_{up}$ and $N_{down}$, and CC is used to determine the genes to include in the respective *Essential Gene sets*, $EG_{up}$ and $EG_{down}$. Only the nodes with CC values less or equal to the computed average CC (e.g., $CC(n_i)\le CC_{avg}(N)$) were included in the respective $EG_{up}$ or $EG_{down}$ gene set.

All six data sets contain duplicate genes that must be removedso they do not compromise the analysis. In many investigations, researchers manually remove the duplicate genes through some customized scripts. This long, tedious, and error-prone process introduce biases, and potentially entangle the PEA results. To overcome this limitation, the proposed preprocessing methodology automatically removes the duplicate genes, and retaining unique genes for further analysis (see Table 2$Tot\#Genes$ columns).

**Table 2:** $Tot\#Genes$ refers to the total number of filtered differential expressed gene from GEO2R tool, after removing duplicate genes

Data sets name	$Tot\#Genes$	$\#Do_{Reg}G$	$\#Up_{Reg}G$	$\#G$
GSE1297	22,283	6	24	30
GSE5281	54,675	185	122	307
GSE16759	54,675	2	5	7
GSE9476	22,283	350	379	729
GSE14924	22,283	124	440	564
GSE24739	54,613	105	69	174

$\#Do_{Reg}G$ is the number of down regulated genes obtained employing the proposed methodology. $\#Up_{Reg}G$ indicates the number of up regulated genes obtained employing the proposed methodology. Finally, $\#G$ is the total number of extracted genes for each data set, holding all criteria

To improve both relevance and specificity of the selected genes within both the $EG_{up}$ and $EG_{down}$ regulated gene sets, each gene was matched to the *IID* network obtained from, filtering out all the unmatched genes. After the mapping on the *IID*, we computed the neighborhoods with distance 1. In this way, the neighborhoods allow identification of new relevant genes exploiting topological information, as reported in Table 3. In order to limit the number of potential genes to use in PEA, all the DEGs holding the following threshold: $Bottleneck(g_i)\ge AvgBottleneck$ were selected. The number of relevant selected DEGs is summarized in Table 4.

**Table 3:** $\#U_{DEGs}$ refers to the total number of filtered out up-regulated DEGs, $\#D_{Reg}G$ is the number of down-regulated genes in each data set

Dataset	$\#U_{DEGs}$	$\#D_{DEGs}$	$\#R_{UDEGs}Ext$	$\#R_{DDEGs}Ext$	$\%Sel_{UDEGs}$	$\%Sel_{DDEGs}$
GSE1297	24	6	2119	1047	0.1077	0.0469
GSE9476	379	350	10,132	11,908	1.7008	0.5343
GSE24739	69	105	4785	5939	0.1263	0.1087
GSE5281	122	185	9113	7013	0.2231	0.1282
GSE16759	5	2	1091	64	0.0091	0.0011
GSE14924	440	124	11,429	4473	0.80473	0.0818

$\#R_{UDEGs}Ext$ indicates the number of up-regulated genes for each gene that successfully mapped onto the IID network. $\#R_{DDEGs}Ext$ represents the down regulated genes for each gene that successfully mapped onto the IID network. Finally, $\%Sel_{DDEGs}$ indicates the percentage of detected relevant genes with respect to the total number of available genes in each data set

**Fig. 4:**
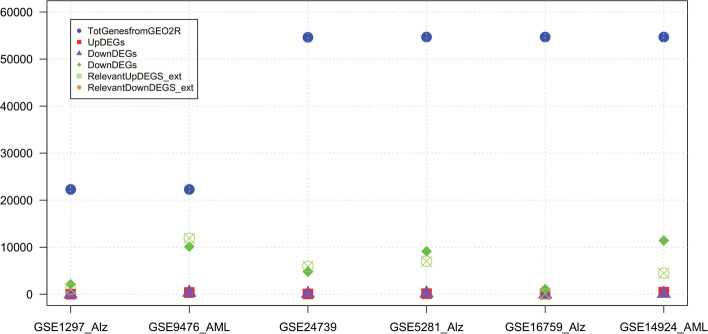
The figure shows the descriptive statistics of six data sets about the Top Gene from GEO2R, Up DEGs, Down DEGs, Relevant Up DEGS, Relevant Down DEGS

**Fig. 5:**
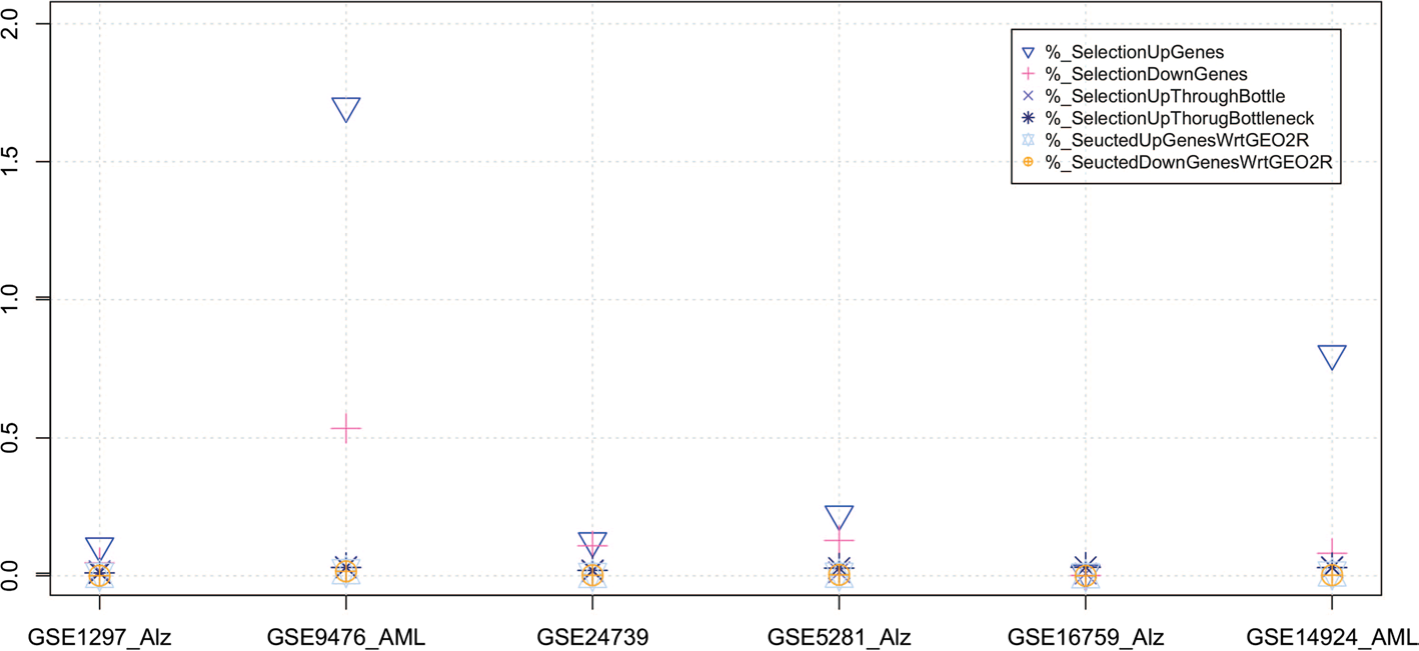
The figure shows the descriptive statistics of six data sets about Selection Up Genes, Selection Down Genes, Selection Up Through Bottle, Selection Up Through Bottleneck Seucted Up Genes Wrt GEO2R, Seucted Down Genes Wrt GEO2R

The descriptive statistics of the six data sets are summarized in Figs. 4 and 5 the number of relevant selected DEGs are listed in Table 4.

**Table 4:** In the table, $\#R_{UDEGs}Ext$ indicates the number of up regulated genes for each gene for it which was possible to obtain a mapping and subsequent extension in *IID* network

Dataset name	$\#R{UDEGs}Ext$	$\#R{DDEGs}Ext$	$BS_{UDEGs}$	$BS_{DDEGs}$	$\%BS_{UDEGs}$	$\%BS_{DDEGs}$
GSE1297	2119	1047	37	11	0.0175	0.0105
GSE9476	10,132	11,908	331	360	0.0327	0.0302
GSE24739	4785	5939	77	115	0.0161	0.0194
GSE5281	9113	7013	118	197	0.0129	0.0281
GSE16759	1091	64	9	2	0.0082	0.0313
GSE14924	11,429	4473	368	133	0.0322	0.0297

$\#R_{DDEGs}Ext$ represents the down regulated genes for each gene for which it was possible to obtain a mapping and subsequent extension in *IID* network. $BS_{DDEGs}$ indicates the percentage of detected relevant gene with respect to the total number of available genes in each data set

It is important to mention that the proposed methods limit the number of DEGs in the PEA to elucidate more relevant biological pathways to the condition under investigation. Tables 6,  7, 10 and 11 report the respective enriched pathways for each gene group.

Tables  6,  7,  10 and 11 report the enriched pathways for each genes’ group.

It is worth noting that the gene groups classification e.g., down- and up- regulated gene groups, provides a two advantages. First, it limits the number of possible enriched pathways by employing fewer more specific genes. Second, it highlights which genes affect the underlining biological functions. This aspect is more evident when analysing the enrichment results in Tables 5 (AML) and 9 (AD) from the ungrouped DEGs. In fact, Table 5 contains the same enriched pathways (in a different order) with respect to Tables 6 and 7, and Table 9 contains the same enriched pathways (in a different order) with respect to Tables 10 and 11, further complicating determination of which DEGs underlie biological mechanisms and functions.

Analysing the data contained in Tables 6, 7,  10, and  11, it should be noted that using of down- and up-regulated gene groups provides a better indication of which group of genes are affecting the pathways responsible for the current phenotype with respect to all unclassified genes.

Comparing the enrichment results obtained using the DEGs without preprocessing and the enrichment results obtained by employing the computed essential DEGs, it supports the proposed approach’s effectiveness in identifying crucial DEGs related to the phenotype under investigation. The effectiveness of the proposed method in selecting proper DEGs is indicated by the obtained from the enrichment’s statistical function, e.g., *Hyper-geometric* function *p* values for each enriched pathway. In fact, higher *p* values refer to more specific biological pathways for the condition under investigation. For example, in Table 6, the first enriched pathway is *Signaling by Interleukins*, a very well-known pathway involved in the generation of AML [37]. In Table 7, the first enriched pathway is *Hemostasis*, a well-known pathway involved in the development and progression of AML [38]. Whereas in Table 8, all ten enriched pathways are generic pathways that do not provide any additional information to the researchers about the DEGs and their involvement in AML. This shows the DEGs role in the PEA. In this enrichment, the *Signaling by Interleukins* pathway is shifted to the 50*th* position in the enrichment ranking, while the *Hemostasis* pathway has been moved to 30*th* position. These results highlight the importance of the chosen DEGs for the PEA. Many DEGs provides many general enriched pathways, challenging researchers to obtain new clues about the relationship between DEGs and biological functions. Finally, it is worthy noting that the use of gene groups along with the identification of essential genes, makes it straightforward to understand which genes are responsible for affecting the underlying biological functions, by linking DEGs with more specific pathways. More information about the other investigated GSE data sets are reported in Additional file 1.

**Table 5:** The first 10 enriched pathways using the whole list of relevant DEGs obtained from the AML GSE24739 data set without using DEG group classification

PathwayName	Pvalue	FDRC	BonfC	$|lg_{2}(P_{value})|$	$|lg_{2}(FDR(P_{value})|$	$|lg_{2}Bonf(P_{value})|$
(1) Signaling pathways	5.29E−13	4.87E−10	4.87E−10	30.94	30.94	30.94
(2) Hemostasis	5.36E−13	2.47E−10	4.93E−10	31.92	30.92	30.92
(3) Developmental biology	9.86E−10	3.02E−07	9.07E−07	21.66	20.07	20.07
(4) Signaling by interleukins	1.37E−09	3.15E−07	1.26E−06	21.60	19.60	19.60
(5) Cell surface interactions at the vascular wall	1.94E−09	3.56E−07	1.78E−06	21.42	19.10	19.10
(6) Cytokine signaling in immune system	5.40E−09	8.28E−07	4.97E−06	20.20	17.62	17.62
(7) Muscle contraction	9.65E−09	1.27E−06	8.88E−06	19.59	16.78	16.78
(8) Signaling by GPCR	1.83E−08	2.10E−06	1.68E−05	18.86	15.86	15.86
(9) GPCR downstream signalling	2.09E−08	2.14E−06	1.93E−05	18.83	15.66	15.66
(10) Cardiac conduction	2.12E−08	1.95E−06	1.95E−05	18.97	15.65	15.65

The *FDRC* indicates the corrected *p* value using *FDR* statistical corrector. The *BonfC* represents the corrected *p* value using *Bonferroni* statistical corrector. Finally, the last three columns contain the $|lg_{2}(\cdot )|$ of each *p* value, for ease comparison

**Table 6:** The first 10 enriched pathways using the list of relevant down regulated DEGs obtained from the AML GSE24739 data set

PathwayName	Pvalue	FDRC	BonfC	$|lg_{2}(P_{value})|$	$|lg_{2}(FDR(P_{value})|$	$|lg_{2}Bonf(P_{value})|$
(1) Signaling by interleukins	8.61E−15	8.21E−12	8.21E−12	46.72	36.83	36.83
(2) Adaptive immune system	1.66E−14	7.92E−12	1.58E−11	45.78	36.88	35.88
(3) Signaling pathways	3.88E−14	1.23E−11	3.70E−11	44.55	36.24	34.65
(4) Cell surface interactions at the vascular wall	1.44E−13	3.44E−11	1.38E−10	42.65	34.76	32.76
(5) Cytokine signaling in immune system	1.48E−13	2.81E−11	1.41E−10	42.62	35.05	32.73
(6) POU5F1 (OCT4), SOX2, NANOG activate genes related to proliferation	4.47E−13	7.11E−11	4.27E−10	41.02	33.71	31.13
(7) Signaling by EGFR	4.47E−13	6.10E−11	4.27E−10	41.02	33.93	31.13
(8) Developmental biology	5.86E−13	6.99E−11	5.59E−10	40.63	33.74	30.74
(9) Hemostasis	5.88E−13	6.24E−11	5.61E−10	40.63	33.90	30.73
(10) Transcriptional regulation of pluripotent stem cells	6.67E−13	6.36E−11	6.36E−10	40.45	33.87	30.55

The *FDRC* indicates the corrected *p* value using *FDR* statistical corrector. The *BonfC* represents the corrected *p* value using *Bonferroni* statistical corrector. Finally, the last three columns contain the $|lg_{2}(\cdot )|$ of each *p* value, for ease comparison

**Table 7:** The first 10 enriched pathways using the list of relevant up regulated DEGs obtained from the AML GSE24739 data set

PathwayName	Pvalue	FDRC	BonfC	$|lg_{2}(P_{value})|$	$|lg_{2}(FDR(P_{value})|$	$|lg_{2}Bonf(P_{value})|$
(1) Hemostasis	1.82E−11	1.60E−08	1.60E−08	35.68	25.89	25.89
(2) Signaling pathways	4.32E−10	1.91E−07	3.81E−07	31.11	22.32	21.32
(3) Metabolism of proteins	4.09E−08	1.20E−05	3.61E−05	24.54	16.34	14.76
(4) Muscle contraction	4.13E−08	9.11E−06	3.64E−05	24.53	16.74	14.74
(5) Cell cycle, mitotic	1.18E−07	2.09E−05	1.04E−04	23.01	15.55	13.23
(6) Mitotic G1 phase and G1/S transition	1.18E−07	1.74E−05	1.04E−04	23.01	15.81	13.23
(7) Cardiac conduction	1.55E−07	1.95E−05	1.36E−04	22.62	15.65	12.84
(8) Cell Cycle	1.78E−07	1.96E−05	1.57E−04	22.42	15.64	12.64
(9) Nervous system development	2.29E−07	2.24E−05	2.02E−04	22.06	15.45	12.28
(10) Amino acid and derivative metabolism	2.88E−07	2.54E−05	2.54E−04	21.73	15.26	11.94

The *FDRC* indicates the corrected *p* value using *FDR* statistical corrector. The *BonfC* represents the corrected *p* value using *Bonferroni* statistical corrector. Finally, the last three columns contain the $|lg_{2}(\cdot )|$ of each *p* value, for ease comparison

**Table 8:** The first 10 enriched pathways using all genes obtained from GEO2R enclosed in the AML GSE24739 data set, without using the proposed pre-processing method

PathwayName	Pvalue	FDRC	BonfC	$|lg_{2}(P_{value})|$	$|lg_{2}(FDR(P_{value})|$	$|lg_{2}Bonf(P_{value})|$
(1) Signaling pathways	7.67E−303	3.01E−301	1.92E−299	1003.606	998.313	992.313
(2) Generic transcription pathway	7.67E−303	3.01E−301	1.92E−299	1003.606	998.313	992.313
(3) Gene expression (transcription)	7.67E−303	3.01E−301	1.92E−299	1003.606	998.313	992.313
(4) SLC-mediated transmembrane transport	7.67E−303	3.01E−301	1.92E−299	1003.606	998.313	992.313
(5) Cellular responses to stimuli	7.67E−303	3.01E−301	1.92E−299	1003.606	998.313	992.313
(6) Cellular responses to stress	7.67E−303	3.01E−301	1.92E−299	1003.606	998.313	992.313
(7) Cellular senescence	7.67E−303	3.01E−301	1.92E−299	1003.606	998.313	992.313
(8) DNA damage-telomere stress induced senescence	7.67E−303	3.01E−301	1.92E−299	1003.606	998.313	992.313
(9) Carbohydrate metabolism	7.67E−303	3.01E−301	1.92E−299	1003.606	998.313	992.313
(10) Metabolism	7.67E−303	3.01E−301	1.92E−299	1003.606	998.313	992.313

.The *FDRC* indicates the corrected *p* value using *FDR* statistical corrector. The *BonfC* represents the corrected *p* value using *Bonferroni* statistical corrector. Finally, the last three columns contain the $|lg_{2}(\cdot )|$ of each *p* value, for ease comparison

**Table 9:** The first 10 enriched pathways using the whole list of relevant DEGs obtained from the Alzheimer GSE16759 data set without using genes’ group classification

PathwayName	Pvalue	FDRC	BonfC	$|lg_{2}(P_{value})|$	$|lg_{2}(FDR(P_{value})|$	$|lg_{2}Bonf(P_{value})|$
(1) Post-translational protein modification	8.20E-05	0.0390	0.0390	4.68	4.68	4.68
(2) Metabolism of proteins	1.67E−04	0.0396	0.0793	4.66	3.66	3.66
(3) Neurophilin interactions with VEGF and VEGFR	7.62E−04	0.1208	0.3625	3.05	1.46	1.46
(4) Disease	0.0013	0.1574	0.6299	2.67	0.67	0.67
(5) VEGF binds to VEGFR leading to receptor dimerization	0.0028	0.2679	1	1.90	0.00	0.00
(6) VEGF ligand–receptor interactions	0.0028	0.2233	1	2.16	0.00	0.00
(7) Signaling by VEGF	0.0029	0.1976	1	2.34	0.00	0.00
(8) Synthesis of 5-eicosatetraenoic acids	0.0047	0.2827	1	1.82	0.00	0.00
(9) Signaling by receptor tyrosine Kinases	0.0047	0.2534	1	1.98	0.00	0.00
(10) Synthesis of leukotrienes (LT) and Eoxins (EX)	0.0049	0.2352	1	2.09	0.00	0.00

The *FDRC* indicates the corrected *p* value using *FDR* statistical corrector. The *BonfC* represents the corrected *p* value using *Bonferroni* statistical corrector. Finally, the last three columns contain the $|lg_{2}(\cdot )|$ of each *p* value, for ease comparison

**Table 10:** The 5 enriched pathways using the list of relevant down regulated DEGs obtained from the Alzheimer GSE16759 data set

PathwayName	Pvalue	FDRC	BonfC	$|lg_{2}(P_{value})|$	$|lg_{2}(FDR(P_{value})|$	$|lg_{2}Bonf(P_{value})|$
1) Synthesis of 5-eicosatetraenoic acids	0.002	0.013	0.735	6.23	0.45	0.45
2) Synthesis of leukotrienes (LT) and eoxins (EX)	0.002	0.014	0.764	6.20	0.39	0.39
3) HIV transcription initiation	0.003	0.026	1.000	5.28	0.00	0.00
4) RNA Polymerase II HIV promoter escape	0.003	0.025	1.000	5.30	0.00	0.00
5) Transcription of the HIV genome	0.005	0.037	1.000	4.76	0.00	0.00

The *FDRC* indicates the corrected *p* value using *FDR* statistical corrector. The *BonfC* represents the corrected *p* value using *Bonferroni* statistical corrector. Finally, the last three columns contain the $|lg_{2}(\cdot )|$ of each *p* value, for ease comparison

**Table 11:** The 6 enriched pathways using the list of relevant up regulated DEGs obtained from the Alzheimer GSE16759 data set

PathwayName	Pvalue	FDRC	BonfC	$|lg_{2}(P_{value})|$	$|lg_{2}(FDR(P_{value})|$	$|lg_{2}Bonf(P_{value})|$
1) PERK regulates gene expression	4.81E−05	0.02	0.02	14.34	5.42	5.42
2) Signaling pathways	1.10E−04	0.03	0.05	13.15	5.23	4.23
3) Unfolded protein response (UPR)	3.33E−04	0.05	0.16	11.55	4.21	2.63
4) Neurophilin interactions with VEGF and VEGFR	0.0011	0.14	0.56	9.77	2.85	0.85
5) ATF6 (ATF6-alpha) activates chaperone genes	0.0028	0.28	1.00	8.45	1.85	0.00
6) ATF6 (ATF6-alpha) activates chaperones	0.0034	0.28	1.00	8.19	1.85	0.00

The *FDRC* indicates the corrected *p* value using *FDR* statistical corrector. The *BonfC* represents the corrected *p* value using *Bonferroni* statistical corrector. Finally, the last three columns contain the $|lg_{2}(\cdot )|$ of each *p* value, for ease comparison

## Discussion

In order to place long lists of differential genes into the context of biological processes and pathways, enrichment pathway analysis is widely used. In this work we proposed a new statistical network pre-processing method to improve the relevance and significance of the DEGs or SNPs of interest when performing PEA attempting to incorporate pathway topology information into the analysis.

Although PEA is an essential part of DEG data analysis, the absence of suitable standards force validation of enrichment results. The following is a literature review for the results from our new statistical network pre-processing methodology.

Analysis of the enriched pathways using the up-regulated relevant DEGs obtained from the AML data set with identifier GSE24739, indicates that the *Signaling by Interleukins* pathway can promote the generation of AML as reported in [37]. *Hemostasis* is a well-known pathway involved in the development and progression of AML [38]. Since both up- and down-regulated gene sets are involved in the *Hemostasis* pathway, all the enriched pathways capture this information. On the other hand, analysis that does not incorporate gene groups, makes it difficult to understand which group of genes are affecting the *Hemostasis* pathway. The third enriched pathway in Table 5 which does not use the gene, groups is the *Developmental Biology* pathway and its relationship with AML as described in [39]. However, the third enriched pathway in Table 7 is the *Muscle contraction* pathway, whose role in AML is explained in [40].

The role of the *signaling pathways* family in the progression and developing of AML is well-known in the literature. In fact, the enriched pathways in Table 6 highlight this peculiarity of enriching the following signalling pathways: *Cytokine Signaling in Immune system*[41], *POU5F1 (OCT4), SOX2, NANOG activate genes related to proliferation* [42], *Signaling by EGFR* [43], and *Signaling Pathways* [44]. The last enriched pathway in Table 6 is *Transcriptional regulation of pluripotent stem cells* whose involvement in AML is described in [45].

Analysis of the enriched pathways in Table 7 reveals the link between the *Metabolism of proteins* and AML as described in [46]. The role of *Amino acid and derivative metabolism* in developing of AML is discussed in [47]. The relation between AML and *Cell Cycle* and *Cell Cycle Mitotic* are described in [48]. In [49] the role *Mitotic G1 phase and G1/S transition* pathway in AML is introduced. In [50] it is clarified that AML can cause *Cardiac conduction* abnormalities in the elderly. Finally, the connection between the *Nervous system development* pathway and AML is documented in [51].

As proof of concept, Table 8 shows the first 10 enriched obtained using all the genes identifiers within the AML GSE24739 data set. Only one of the enriched pathways seems to have a connection with AML, the *Cellular Senescence* pathway [52]. To the best of our knowledge, we were unable to find any connection between the remaining nine enriched pathways in Table 8 and AML. This shows that using many less specific DEGs provides more enriched pathways but they are disconnected from the biological context of reference.

Thus, it is worth noting that using the improved list of genes provides more relevant enriched pathways as demonstrated in Tables 5, 6, and 7, where all the enriched pathways have a connection with AML. Indeed, using a generic list of genes, the ratio between enriched pathways with the biological context of reference, e.g., AML, drops to $10\%$.

The connection among the first three enriched pathways in Table 9 and Alzheimer’s are the following. The association between the *Post-translational protein modification* pathway and AD is reported in [53]. The relationship between the *Metabolism of proteins* pathway and AD is provided in [54]. While in [55], the authors describe the role of the *Neurophilin interactions with VEGF and VEGFR* pathway and AD.

Searching the scientific literature, we find the following connection between the first three enriched pathways in Table 10 and AD. The role of the *Synthesis of 5-eicosatetraenoic acids* and *Synthesis of Leukotrienes (LT) and Eoxins (EX)* pathways with the AD is reported in [56] and [57], respectively. The implication of *HIV Transcription Initiation* pathway in AD is explained in  [58].

Table 11 lists the enriched pathways using the up-regulated relevant DEGs obtained from the *GSE16759* data set. In [59], the authors describe the implication of the *PERK regulates gene expression* pathway with AD. In [60] the authors clarify the implication of *Signaling Pathways* in the development of many human diseases including AD disease. The authors in [61] characterize the association between *unfolded protein response (UPR)* with onset of familial Alzheimer’s disease (Fig. 5).

**Fig. 6:**
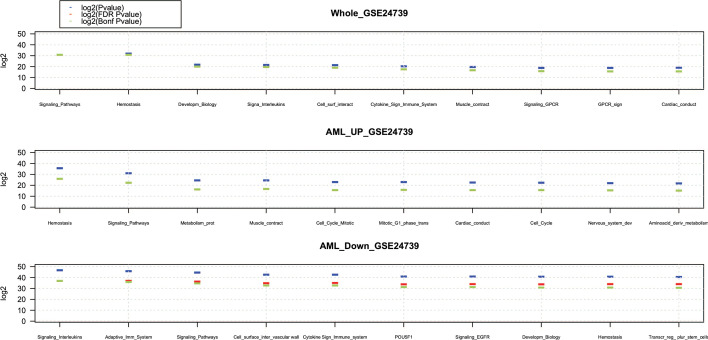
The first 10 enriched pathways using the whole list of relevant DEGs, up-regulated DEGs, and down regulated DEGs obtained from the AML GSE24739. The blue mark represents the $log_{2}($p*values*), the red mark represents $log_{2}(FDR$p*values*), and the green mark represents $log_{2}(Bonferroni$p*values*)

**Fig. 7:**
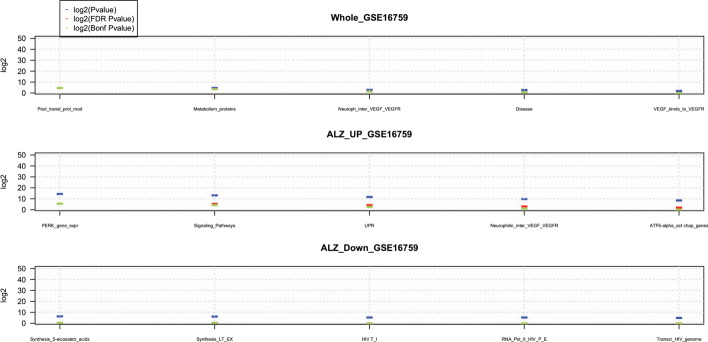
The first 10 enriched pathways using the whole list of relevant DEGs, up-regulated DEGs, down-regulated DEGs obtained from the AD GSE16759. The blue mark represents the $log_{2}($p*values*), the red mark represents $log_{2}(FDR$p*values*), and the green mark represents $log_{2}(Bonferroni$p*values*)

Comparing the enriched pathways in Figs. 6 and 7, further highlights the benefits of the proposed approach, revealing more specific pathways affecting the biological functions and mechanisms.

Finally, we performed PEA using the grouped and ungrouped DEG sets to assess the effectiveness of the proposed DGE preprocessing and selection method. Analyzing the obtained pathway enrichment results using both data sets highlight that DEGs critically impact PEA, since employing an ungrouped DEG set can lead to poor enrichment results. Also, the first ranked enriched pathway using grouped DEGs is related to the condition under investigation, which may induce new biological discoveries and simplify research.

## Conclusions

In this work, we proposed a new statistical network pre-processing approach to identify relevant DEGs that can improve PEA results, helping researchers identify the affected underlying biological functions and processes. The proposed method provides a two-fold improvement. First, network analysis yields fewer DEGs, choosing only relevant DEGs that directly involved with the condition under investigation. Second, the detected DEGs improve the enriched pathways’ statistical significance over a more general list of genes. As a drawback, the number of enriched pathways is still too large; thus, future research should be aimed at developing a method to further reduce the number of enriched pathways.

## Supplementary information

**Additional file 1**: Provides detailed information about the other investigated GSE data sets.

## Data Availability

The data sets used and analyzed in this study are freely available in GEO database. GEO data set links: https://www.ncbi.nlm.nih.gov/geo/query/acc.cgi?acc=GSE1297; https://www.ncbi.nlm.nih.gov/geo/query/acc.cgi?acc=GSE5281; https://www.ncbi.nlm.nih.gov/geo/query/acc.cgi?acc=GSE16759; https://www.ncbi.nlm.nih.gov/geo/query/acc.cgi?acc=GSE9476; https://www.ncbi.nlm.nih.gov/geo/query/acc.cgi?acc=GSE14924; https://www.ncbi.nlm.nih.gov/geo/query/acc.cgi?acc=GSE24739; **Reactome** database link: https://reactome.org/download-data; **KEGG** database link:https://www.kegg.jp; **BiP** software tool link: https://gitlab.com/giuseppeagapito/bip; **GEO2R** software tool link: https://www.ncbi.nlm.nih.gov/geo/geo2r/. Also, all the links to the data sets and materials have been provided through the manuscript.

## Abbreviations

AD: Alzheimer’s disease
AML: Acute myeloid leukemia
API: Application program interface
BioPAX: Biological pathway exchange
BiP: BioPAX-parser
CC: Closeness centrality
CLL: Chronic lymphocytic leukemia
DEG: Differential expressed gene
DNA: Deoxyribonucleic acid
EG: Essential gene sets
FDR: False discovery rate
GEO: Gene Expression Omnibus
GWAS: Genome-Wide Association Studies
HT: High throughput
IID: Integrated interactions database
logFC: Logarithm of fold change
KEGG: Kyoto Encyclopaedia of Gene and Genome
miRNA: micro RNA
mRNA: messenger RNA
NGS: Next generation sequencing
ORA: Over represented analysis
PEA: Pathway enrichment analysis
RNA: Ribonucleic acid
SBML: Systems biology markup language
SM: Similarity matrix
SNPs: Single nucleotide polymorphism
